# A biface production older than 600 ka ago at Notarchirico (Southern Italy) contribution to understanding early Acheulean cognition and skills in Europe

**DOI:** 10.1371/journal.pone.0218591

**Published:** 2019-09-26

**Authors:** Marie-Hélène Moncel, Carmen Santagata, Alison Pereira, Sébastien Nomade, Jean-Jacques Bahain, Pierre Voinchet, Marcello Piperno

**Affiliations:** 1 UMR 7194 HNHP, National Museum of Natural History, Paris, France; 2 UMR 5199 PACEA, University of Bordeaux 1, Bordeaux, France; 3 Ecole française de Rome, Piazza Farnese, Roma, Italy; 4 Laboratoire des Sciences du Climat et de L’Environnement, UMR 8212, LSCE/IPSL, CEA-CNRS-UVSQ, Gif-sur-Yvellet, France; 5 Université Paris-Saclay, Gif-Sur-Yvette, France; 6 Museo archeologico "Biagio Greco", Mondragone, Italy; Max Planck Institute for the Science of Human History, GERMANY

## Abstract

For the past decade, debates on the earliest evidence of bifacial shaping in Western Europe have focused on several key issues, such as its origin (i.e., local or introduced), or on what should define the Acheulean culture. Whatever hypotheses are proposed for its origin, the onset and technological strategies for making Large Cutting Tools (LCTs), including biface production, are key issues and are often associated with other behavioural changes, such as increased core technology complexity. Current archaeological patterns do not support the existence of transitional industries. Rather, the scant evidence suggests that biface production associated with the management of bifacial volume was widespread around 700 ka. Among the earliest sites, the site of Notarchirico in Southern Italy stands out as one of the most significant examples. ^40^Ar/^39^Ar ages and ESR dates recently provided a revised chronology for the whole sedimentary sequence and constrained the archaeological levels between ca. 610 and 670 ka. Five archaeosurfaces (A, A1, B, D and F) yielded LCTs, including bifaces, during Marcello Piperno’s excavations from 1980 to 1995. In light of this new chronological framework, which is much shorter than previously thought, we propose in this contribution a revision of the bifaces by applying the “*chaine opératoire*” method for the first time (analysis of reduction processes). Our goals are to assess biface production in this early Western European locality and to characterize the strategies applied at the site throughout the sequence. A corpus of 32 tools was selected from the A-A1, B, D and F archaeosurfaces. The technological analysis shows that hominins had the capacity to manage bifacial volumes, when raw material quality was adequate. Clear differences do not emerge between the different levels in terms of shaping modes or final forms. However, we demonstrate that the oldest level (level F), with the richest corpus, lacks flint and displays a higher diversity of bifaces. This ability to manage bifacial and bilateral equilibrium, as well as the diversity of the morphological results, is observed in a few penecontemporaneous sites (700–600 ka), both in the north-western and southern parts of Western Europe. These patterns suggest that hominins mastered well-controlled and diversified biface production, combining intense shaping and minimal shaping, and shared a common technological background regardless of the geographical area, and applied this technology regardless of the available raw materials. The degree of skill complexity of hominins in Western Europe between 700 and 600 ka, the current lack of evidence suggesting “gradual industries” between core-and-flake series and Acheulean techno-complexes, raise numerous questions on the origin of new behaviours in Western Europe, their mode of diffusion, and their association with *Homo heidelbergensis* or other Middle Pleistocene populations.

## Introduction

Over the past decades, debates concerning the appearance of biface production in Western Europe have focused on two key issues: 1) its origin (i.e., local or introduced), 2) what should define the Acheulean culture (see for example [1–7]). The onset of bifacial shaping is of course central to these debates, whatever hypotheses are proposed for its origin. It could be related to the earliest evidence of *Homo heidelbergensis* and other European Middle Pleistocene hominins around 700 ka [8], recently associated with other behavioural changes, such as greater core technology complexity [9, 10]. Discoveries made over the past decade offer the opportunity to take a fresh look at European Acheulean techno-complexes. Large Cutting Tools (LCTs), including bifaces, are no longer considered as “*fossiles directeurs*”, as proposed by Gabriel de Mortillet and other prehistorians in the past [11]. However, the presence of the biface is still closely tracked as it remains an iconic Acheulean tool, although it is not present in all Lower Palaeolithic assemblages and increasingly appears to be related to activities and/or types of raw material management and/or traditions [7, 10, 12].

The most recent scientific results on the cradle of the Acheulean in East Africa [13, 14] offer a new interpretation of these techno-complexes and highlight the variability of the heavy-duty component, including LCTs. Indeed, LCTs encompass a wide variety of tools (bifaces, picks and cleavers), made on large flakes (> 10 cm), cobbles, blocks, fragments and others. Technological variability of LCT manufacture is taken into consideration alongside core technology and land use patterns to investigate the cultural attribution of an assemblage and potential technological shifts (for instance, in Africa between the Oldowan and the early Acheulean) (i.e. [14]). In Europe, recent findings question the presence of a technological shift between core-and-flake industries and bifacial industries and tend to focus on the timing of the manufacture of bifacially symmetrical LCTs [2, 12].

Some recent studies document the inter- and intra-site diversity of the earliest LCTs in European assemblages, whose age has been pushed further back in time over this past decade [9, 15, 16].

The discovery of two crudely made Large Cutting tools (LCTs) at La Boella in Spain [17, 18] dated attempts at LCT production to the end of the early Pleistocene (1 Ma- 900 ka), which could be interpreted as either a local evolution or an early introduction. However, no transitional industries have been found so far for that period and elaborate biface production only appears 200 ka later in different regions of Western Europe, for instance at the site of la Noira, located in the centre of France and dated to approximately 700 ka [9, 15]. In this context, “elaborate” refers to the ability to manufacture large tools with bifacial balance and volume, two convergent edges and a tip.

Among the earliest sites with biface production, the site of Notarchirico, located in Southern Italy (Venosa basin, Basilicata), stands out as one of the most compelling examples [19]. The site yielded a well-developed seven-metre sequence of fluvial sediments with eleven archaeological levels, five of which contain tools described as bifaces [19]. 40Ar/39Ar ages and ESR dates have revised the chronology of the whole sedimentary sequence and constrained all archaeological levels between ca. 610 and 670 ka [16] (Table 1).

**Table 1:** ^40^Ar/^39^Ar ages obtained on single K-feldspar grains from the Notarchirico sequence (Venosa, Italy). Age 1 are calculated using ^40^K decay constant of Steiger and Jäger [31] and ACs-2 standard at 1.193 Ma according to Nomade et al. and Pereira et al. [16, 32]. Age 2 are calculated using ACs-2 standard at 1.1891 Ma according to Niespolo et al. [33] (this work). And ^40^K decay constant of Renne et al. [32]. The ages displayed in Pereira et al. [16] were recalculated using the ^40^K decay constant of Renne et al. [34] and the flux standard ACs-2 at 1.1891 Ma. Ages are only marginally changed (-0.3% younger), but the full external uncertainties are much better than previously reported by [16] due to the improved precision of the optimized K total decay constant of Renne et al. [34]. Levels 2–2 and 2–1 are part of the Notarchirico Tephra Complex (NTC), the only primary volcanic deposit of the section. The other ages should be considered as indicating the last volcanic event recorder in the sediment. Note that only unit 3 and level 1–6 top indicate a volcanic event different to those related to the NTC.

Levels dated in our study	Age 1 (ka)	Analytical uncertainties at 2σ (ka)	Full external uncertainties (ka)	Age 2 (ka)	Analytical uncertainties at 2σ (ka)	Full external uncertainties (ka)
level 1–6 top	614	± 4	± 12	612	± 4	± 5
level 1–5	652	± 3	± 12	650	± 3	± 4
level 1–3	660	± 8	± 12	658	± 8	± 9
level 2–6	663	± 3	± 13	661	± 3	± 4
level 2–2	661	± 3	± 14	659	± 3	± 4
level 2–1	660	± 5	± 14	658	± 5	± 6
Unit 3	670	± 5	± 14	668	± 5	± 6

In light of this new chronological framework, we propose here a revision of the bifaces found during Marcello Piperno’s excavations from 1980 to 1995 by applying the “*chaïne opératoire*” method (reduction processes) [20, 21] for the first time to assess biface production at this Western European locality. This study contributes to the discussion on the continuity or transition from non-Acheulean to Acheulean assemblages in Southern and north-western Europe [18].

Current archaeological data suggest that the 700 and 500 ka time period was crucial, with sporadic occupations including new behaviours and biface production [22, 23]. All these changes foreshadow the cultural shift that occurred during the harshest glacial period, known as Marine Isotope Stage 12 (MIS hereafter) [4, 5, 6, 12, 24, 25]. After MIS 12, more abundant archaeological data indicate regionalization throughout Europe with new behaviours, standardization of both bifacial shaping and core technologies, and the development of Neanderthal features [8]. All the early evidence of biface shaping corresponds to the lapse of time between two major cold events, MIS 16 and 12. Some studies have suggested that it can be explained by successive phases of recolonization and the disappearance of small groups of populations as a result of climate fluctuations or the opening of new migration routes [24, 26, 27].

In this general context, this contribution addresses six questions: (1) Was there bifacial management of LCT volume at Notarchirico and are these tools unquestionable bifaces? (2) Were there standardized manufacture processes for making bifaces? (3) Was there intra-site diversity in biface production strategies during and between occupation phases? (4) If so, was intra-site diversity due to the type of stones, blanks, different traditions or types of occupation? (5) Are the strategies recorded at Notarchirico similar to the behavioural variability observed during the same time period (700–600 ka) elsewhere in Western Europe? (6) Can the site contribute to our understanding of the timing and characterization of the earliest Acheulean in Western Europe and its relationship to *Homo heidelbergensis* and Middle Pleistocene populations?

## The site of Notarchirico

**Fig 1:**
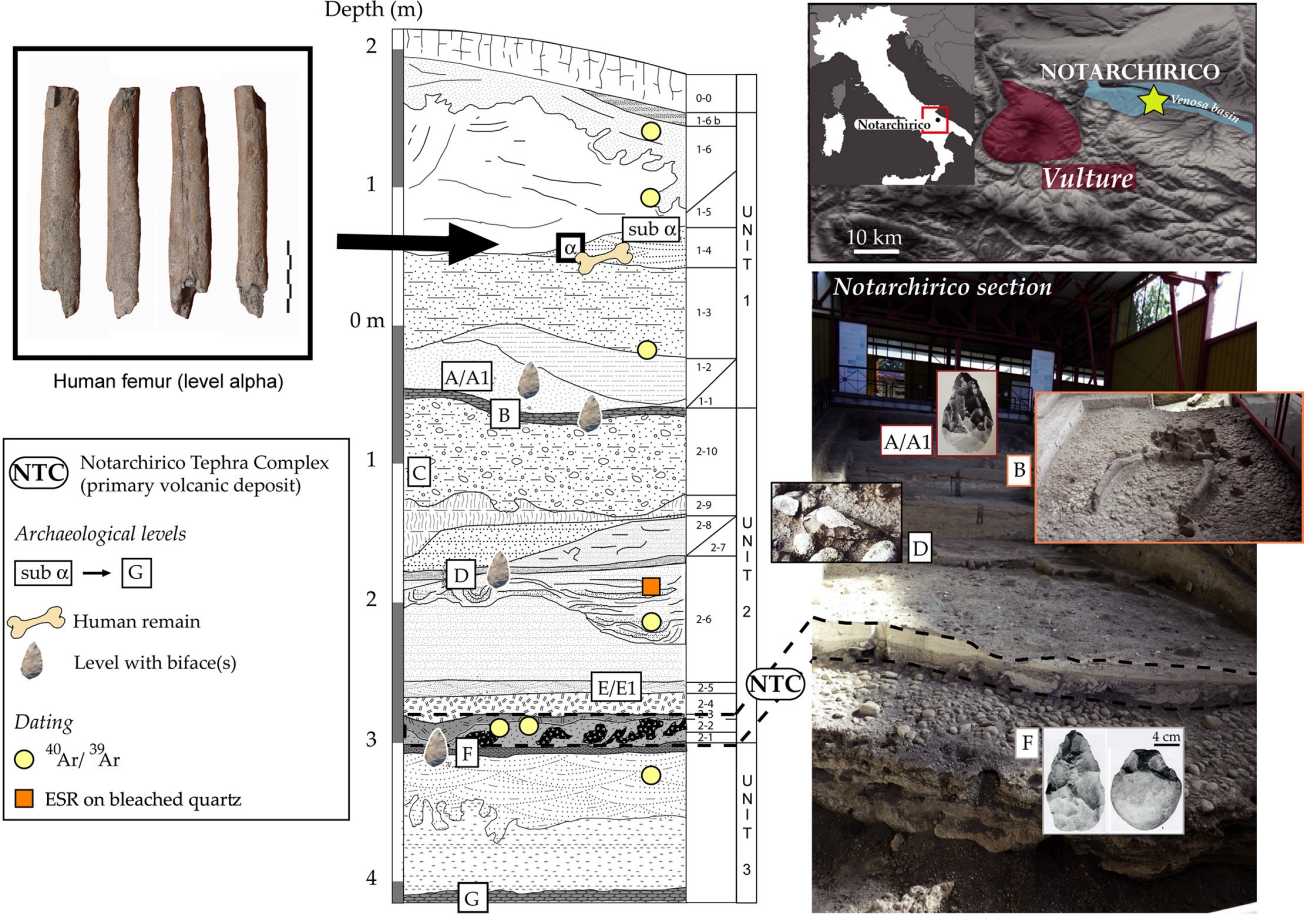
Location, view and description of the stratigraphic sequence of Notarchirico (Venosa, Basilicata)—for further details please refer [16, 28]. Photos M-H. Moncel, C. Santagata, A. Pereira, M. Piperno; drawings A. Pereira.

The archaeological site of Notarchirico is located close to the city of Venosa (Basilicata), 300 km southeast of Rome in Italy (Fig 1). During excavations by M. Piperno in the 1980s, eleven archaeosurfaces were discovered in a seven-metre thick fluvial-derived sedimentary sequence rich in volcanic materials from the neighbouring Monte Vulture stratovolcano (Fig 1B) [28]. A recent geochronological study including ^40^Ar/^39^Ar and ESR ages constrains the occupation period of the site, spanning the entire MIS 16 glacial stage ([16] modified) (Table 1). The infill records recurrent periods of occupation, most of which are interpreted as butchery activities, although the association between artefacts and bones is not clearly demonstrated, as illustrated by the “Elephant Butchery area” at the top of the sequence (archaeosurfaces A-A1-B) (Fig 1). The faunal assemblage, whose main species are *Elephas antiquus*, *Dama clactoniana*, *Bos primigenius* and *Bison schoetensacki*, is attributed to the Ponte Galeria faunal unit. The palynological results obtained in several levels point to a predominantly open and cold environment [19]. A fragment of a human femur, attributed to *Homo heidelbergensis* [29], was discovered in the upper level supra-α (Fig 1).

The extension of the excavated areas varies between 133 m^2^ down to 19 m^2^, depending on the different archaeological levels. The archaeosurfaces consist of more or less dense beds of pebbles of limestone, secondary quartz and quartzite, corresponding to lakeshore remains. The materiel was found among beds of pebbles and flint, where quartz and quartzite artefacts are better preserved than limestone. Most of the lithic material is composed of a heavy-duty component and smaller elements derived from core technology (cores and flakes). The largest tools are mainly made in limestone and quartzite. Quartz, quartzite, limestone and flint are used for the debitage. Hominids used local pebbles/cobbles and small flint nodules, probably collected from rivers and/or lakeshores on or near the site at the time of occupation (*i*.*e*., mainly MIS 16). The heavy-duty component is characterized by diversified but poorly-standardized artefacts and includes unifacial and bifacial pebble tools, some of which are pointed, and rare pseudo-cleavers on limestone pebbles [19]. Small nodules or pebbles were used for the debitage, and knapped by freehand or bipolar percussion, on one or multiple surfaces with unipolar or convergent removals [30]. Some cores are discoid-like with alternate debitage but no platform preparation. In many cases, backs are preserved. There are few flake scars and these are frequently hinged. Raw material shape clearly influenced core technology, especially for limestone. Flint cores are more exhaustively depleted and then broken. End-products are often small (< 15–20 mm in flint) with rare larger flakes, retouched or unretouched, mainly in limestone, quartz or quartzite (50–100 mm). Flint cores range in size from 20 to 40 mm and some limestone pebble tools may be chopper-cores or cores (50–200 mm long). We note that retouch substantially modifies initial blank shape in many cases (scrapers, notches, denticulates and Tayac points).

## Material and methods

### Lithic material

**Fig 2:**
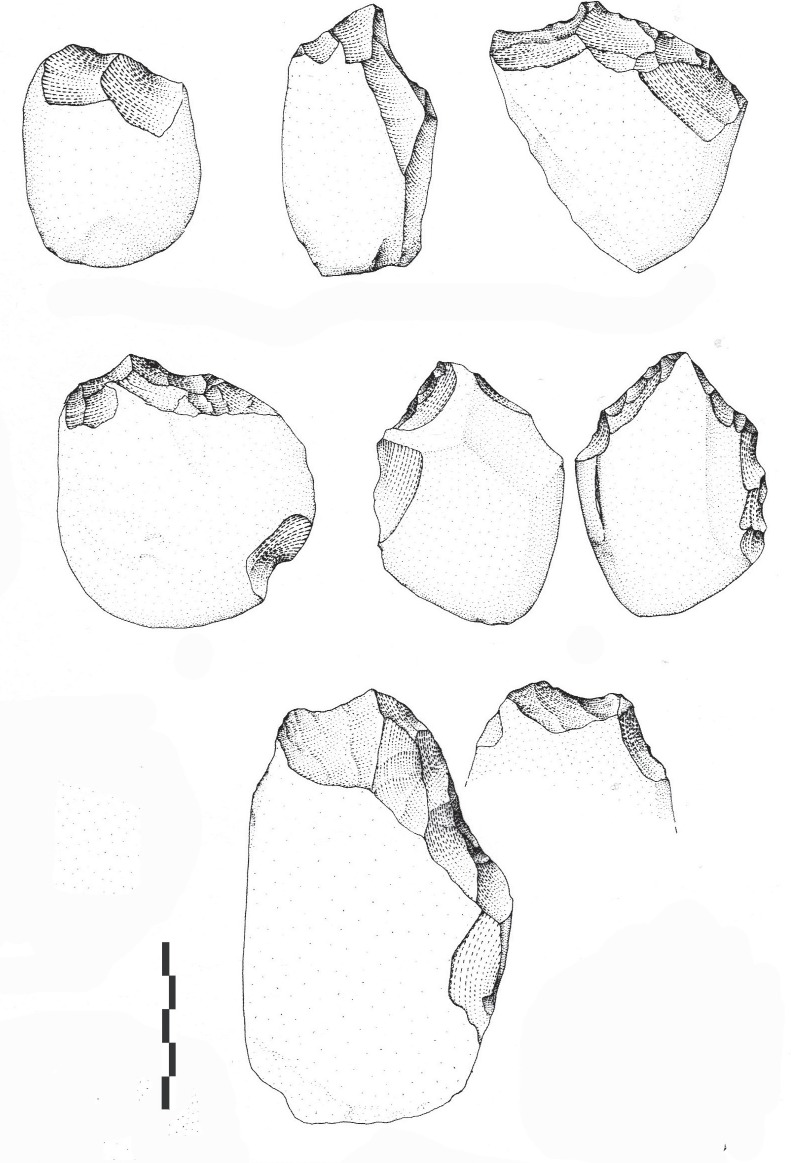
Notarchirico Archaeosurface A. Examples of pebble tools in limestone. Unifacial and partial bifacial shaping. (drawings M. Pennachioni).

Hereafter, our work focuses on the material found during the excavations directed by M. Piperno [19]. The heavy-duty component is mainly composed of unifacial pebble tools and secondary bifacial pebble tools (Table 2). Unifacial pebble tools in limestone, with a convex or rectilinear cutting edges, dominate the corpus of pebble tools. Pointed pebble tools total between 10 and 40% of the pebble tools series (Fig 2). Pebble tools are distinguished by the limited number and extension of the removals, on one edge of the extremity of the pebble/cobble. Shaping does not cover more than a third or half of one surface of the pebble, mainly a small part of the surface of the pebble/cobble. Among this heavy-duty component, five archaeosurfaces yielded tools referred to as bifaces (A, A1, B, D and F, Table 2).

**Table 2:** Number of bifacial Large Cutting Tools at Notarchirico (M. Piperno excavations) and additional quantitative data on the lithic series.

		Bifaces and bifacial tools			Cleavers-like on pebble	Pebble tools	Total series
Levels or archaeosurfaces	Extension excavated area	Flint and siliceous rocks	Limestone	Quartzite	Limestone	All stones	
A-Alpha- Base A1	133 m^2^	3	5	-	-	124	348
B	32 m^2^	6	5		2	94	107
C	20 m^2^	-	-	-	-	74	78
D	25 m^2^	1	1	-	-	29	300
E–E1	20 m^2^	-	-	-	-	20	614
F	19 m^2^	-	7	2	-	5	30

**Fig 3:**
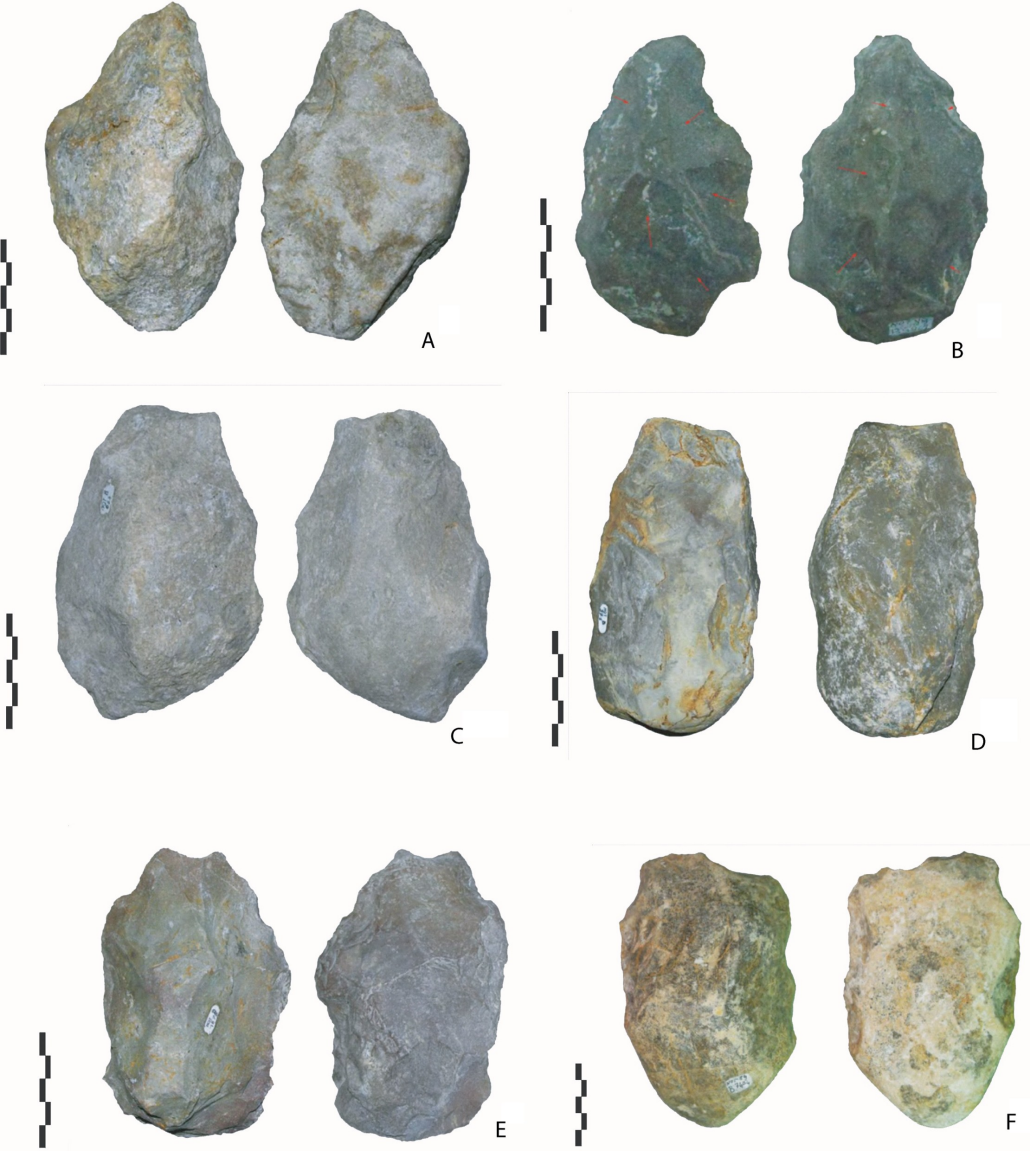
Notarchirico Archaeosurface B. Crude bifacial tools (n°A, B, C, E) and a cleaver-like tool on a pebble (n°D, F) (photos C. Santagata).

All the material (n = 1387 including the 29 bifaces from this study) is stored at the Venosa Museum (Museo archeologico nazionale di Venosa, Basilicata region, Italy) except the three bifaces in limestone badly preserved of level F, which are in situ on the site of Notarchirico. The specific materials analyzed in this study are described in detail in the Materials and Methods section. Qualified, interested may submit queries related to access to the Soprintendenza of Basilicata (Italy, email = sabap-bas.archeopz@beniculturali.it). The authors do not have special access privileges to these materials and accessed them in the manner described. The site of Notarchirico (40°58’08.33”N– 15°53’17.70’E) is located at Venosa. All the material from each level of the sequence, stored at the Venosa Museum (Basilicata region) and *in situ* on the site (for level F), was observed. Our study corpus totals 32 bifaces or LCTs, comprising between 2 and 11 tools per level in various raw materials (flint, limestone and quartzite). The slight differences in the number of bifaces published by Piperno (1999) and this analysis is due to our revision of the collections. For the selection of our corpus, we took into account the extension of shaping on both faces, or at least on one face, and the purpose of the manufacture. For this study, we did not take into account one or two cleaver-like tools on pebbles (level B) (Fig 3). The tools on a quadrangular pebble with partial peripheral shaping can be considered as a cleaver-like tool (150–160 mm long). We did not take in account as well a broken extremity of a possible bifacial tool (level F) due to the bad preservation.

### Methods: Documenting LCT technology, shape and size variability

Our analysis aims to define the technological strategies and reduction processes used to produce our corpus of LCTs in each level and raw material, the impact of raw materials and blank geometry, based on [14, 20, 35–42]. We describe the different production methods and tool variability in the final shaping phase. For fully-shaped tools, it was not possible to describe the previous shaping phases. We focus on the detailed description of the best-preserved tools. The application of statistical methods is limited due the small number of artefacts, but box-plots and graphs help compare tool size per level and raw material.

We analysed the following aspects of bifacial technology, for each raw material type:

(1) The geometry and identification of the blank when possible (pebble or flake), the number, size and organization of removals (centripetal, crossed, unipolar), number of series of removals (large removals for initial shaping, then smaller removals for finishing volume management and final removals for the cutting edges), extension and organization of shaping on both faces (covering or not the whole surfaces of the tool), secondary retouch and location (final retouch on the cutting edges and the tip to thin the tool and rectify the edges), shape of the cutting edges (rectilinear or sinuous), shape (round, pointed, transversal) and type of tip management (as part of the whole shaping of the tool, independently of and before or after general shaping with specific attention to the tool extremity). The number and organization of shaping phases are identified by figures (for instance 1.1, 1.2) to define how volume was managed during shaping. The identification of the use of soft/hard hammers is based on the shape of the scars on the tools—depth and invasiveness see [9] -, and on the identification of curved flakes with a lip in the flake assemblage,

(2) The morphological results of shaping (shape, section, bilateral and bifacial symmetry of the tools, shape of the apical part and base). Bilateral symmetry is determined by measuring each side of the tool in relation to the position of the central line passing through the tip. Bifacial symmetry is determined by measuring the thickness of each face from the surface to the cutting edge,

(3) Quantitative data (maximum length, width and thickness, and location of the maximum thickness, cutting edge angles when measurable all around the periphery).

Few small scars were identified as taphonomical and post-depositional damage. The smooth aspect of some limestone tool edges is due to chemical processes during slow geological processes on lakeshores, as demonstrated in [19, 28].

The following characteristics were taken into consideration for defining a biface and a biface production: (1) bifacial shaping aiming to produce a global volume, (2) bifacial and bilateral equilibrium, (3) face-by-face or alternate management with one or several series of removals, and sometimes final retouch of the cutting edges; (4) the presence of two convergent edges and a tip (rounded or pointed) with specific management, and (5) the minimal extension of shaping related to volume management.

## Results

### Description of biface production sequences and morphology at Notarchirico

#### Archaeosurfaces A, base A1, top B (Elephant area) (n = 8)

**Fig 4:**
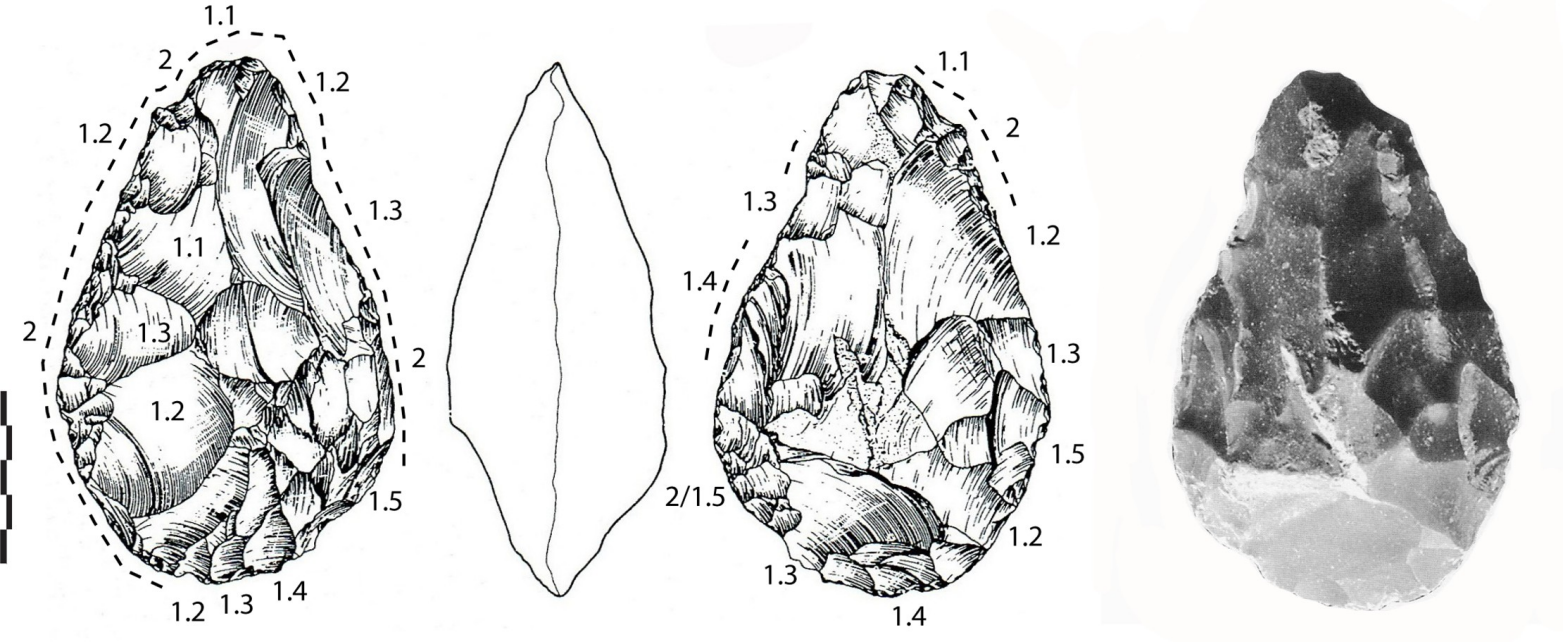
Notarchirico Archaeosurface A1. Flint biface. Dotted line: final removals and retouch. White arrows: direction of the main removals for managing bifacial volume. The numbers indicate shaping phases and sub-phases. (drawings M. Pennachioni, modified; photo L. di Masi).

**Fig 5:**
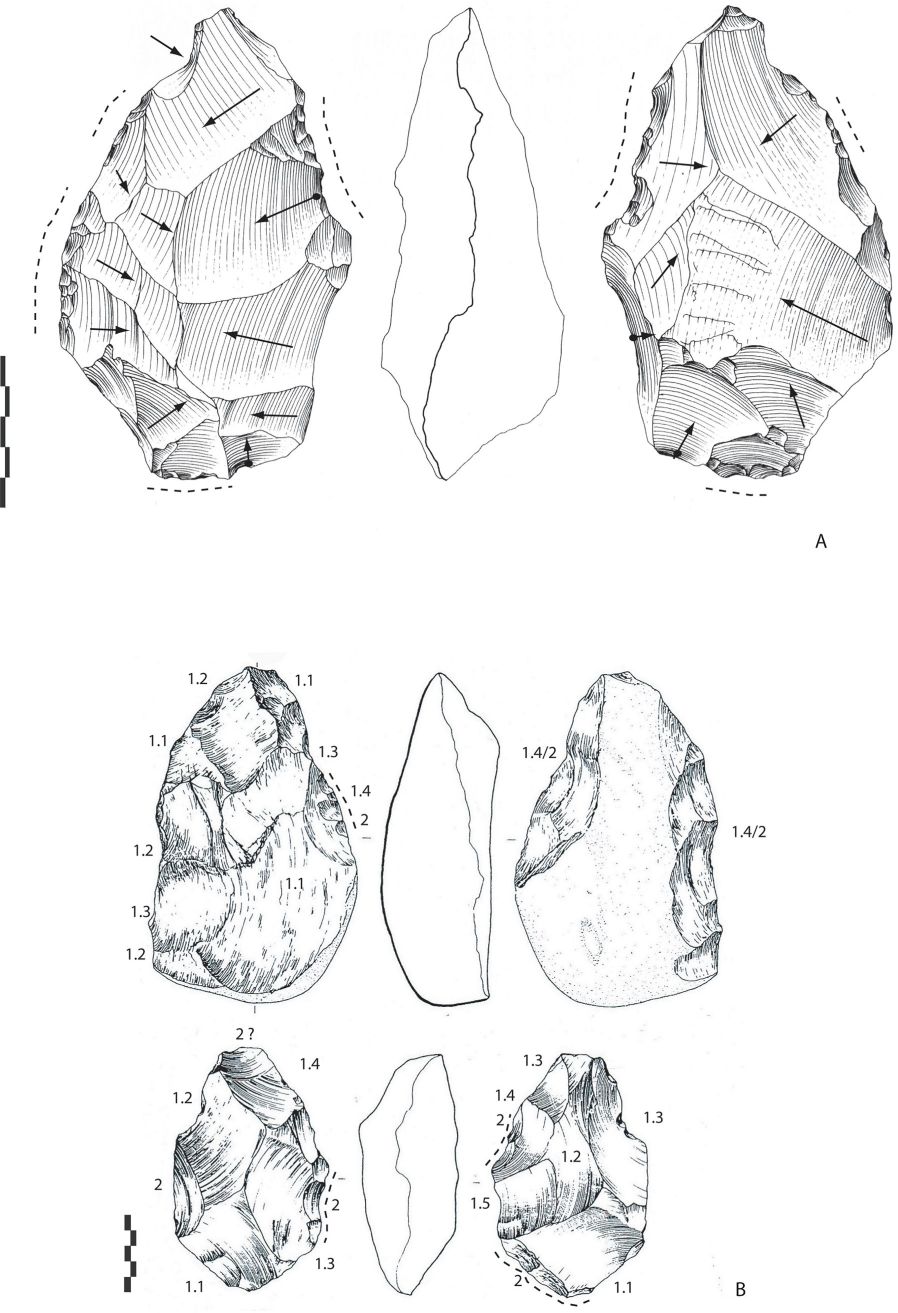
Notarchirico A. Archaeosurface A. Limestone biface. B. Archaeosurface A1. Biface in limestone and biface in siliceous limestone. Dotted line: final removals or retouch. The numbers indicate shaping phases and sub-phases (drawings G. Marchesi, modified).

The flint LCTs (n = 3). The flint bifaces (120-68-51 mm; 165-112-71 mm) are shaped by numerous invasive and centripetal removals by alternate shaping, at least for the final shaping phase (Figs 4 and 5), which totally cover both faces. Shorter removals (some hinged) cover the edges, which are regular for one piece (155-98-72 mm). They are located all around the tool on both faces. Some retouch is visible in places on the periphery of one tool on both faces. Retouch and short removals are part of the bifacial balance management of the tool and regularize cutting edges and/or finish the shaping. The angles vary on the periphery between 75 and 85°. The tip of the tools is pointed and is shaped as part of general tool management, probably during the first phases of manufacture, in the same way as for the base of the tool. The thickest part of the tool is the middle part. The cross-section is symmetrical for one tool (Fig 3). Blank types are unknown due to the invasiveness of shaping.

Limestone LCTs (n = 5). Limestone pebbles are of two types, siliceous and marly. Two of the five bifaces made of siliceous limestone (Fig 5B) are shaped in a similar way to the marly limestone tools with large removals covering the faces (less than 10 removals per face). The flint tools, however, were shaped with a series of smaller removals. The limestone bifaces are symmetrical in shape and section. Sporadic retouch (or short removals) is visible on some areas of the edges to finish shaping. The tip is pointed or broken (during use or due to a transversal removal, Fig 4 bottom, 120-70-50 mm). The angles of the cutting edges are similar to those of flint bifaces.

For two pieces (150-99-60 mm; 165-98-45 mm), the blank is a cobble or a half cobble with a thickness varying between 45 and 60 mm and biconvex surfaces. Shaping consists of a few invasive removals (less than 10 for each face), indicating minimal shaping, in general with more removals on one face than the other (Fig 5A). Shaping is alternate, and the management of both faces is linked to the available angles. The tools are unsymmetrical (bifacial and bilateral). The tip is frequently rounded and its shaping is part of overall tool shaping. A notch accentuates the “déjetée”/offset apical part and invasive lateral removals (Fig 5).

#### Archaeosurface B (n = 11)

Crude bifacial tools? (n = 6). The corpus is composed of three tools in flint and three in limestone (Fig 3). Tool shape is irregular. All the tools are made by few invasive removals, with one face being more extensively shaped (from 50% to 90% of the surface). The second face remains cortical and flat. Except for one piece, there is no second series of removals and final retouch. Shaping is alternate or face-by-face. The tools are asymmetrical in shape and cross-section. They measure between 100 and 150 mm, and all are on pebbles.

**Fig 6:**
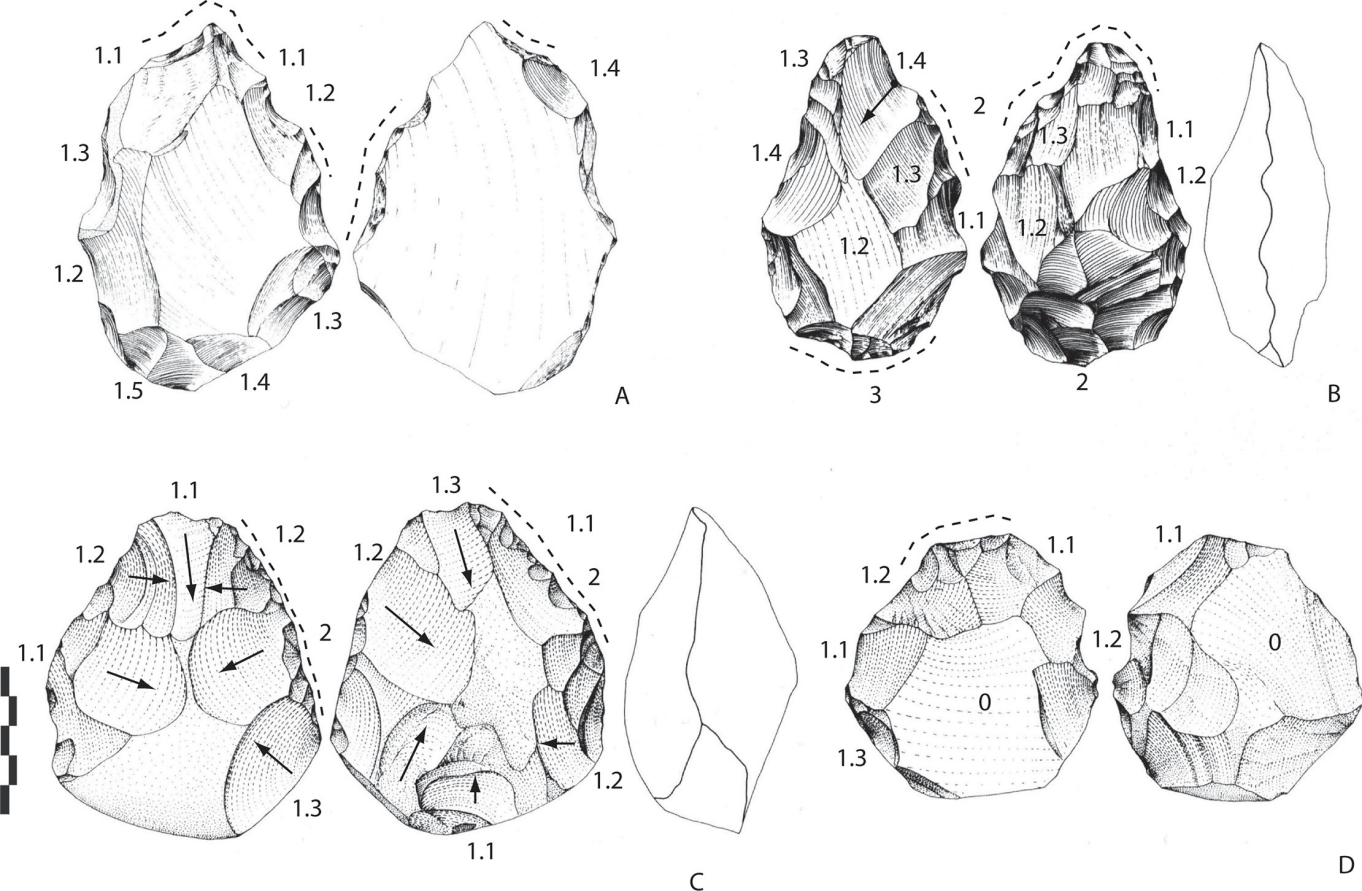
Notarchirico Archaeosurface B. Flint bifaces (A, B) and limestone bifaces (C, D). Dotted line: final removals or retouch. The numbers indicate shaping phases and sub-phases. (drawings M. Pennachioni, modified).

Flint LCTs (n = 3). One tool is totally shaped by large and small removals (131-90-45 mm), first by invasive and centripetal removals and then by shorter bifacial removals, especially on the round tip and the base (Fig 6A and 6B). Shaping is alternate. The tool is symmetrical and the tip is slightly “déjetée”, due to a final large removal and short unifacial removals/retouch. The aspect and angle of one large removal in the middle of one face raises the question of the possible use of a large flake or broken nodule/cobble.

**Fig 7:**
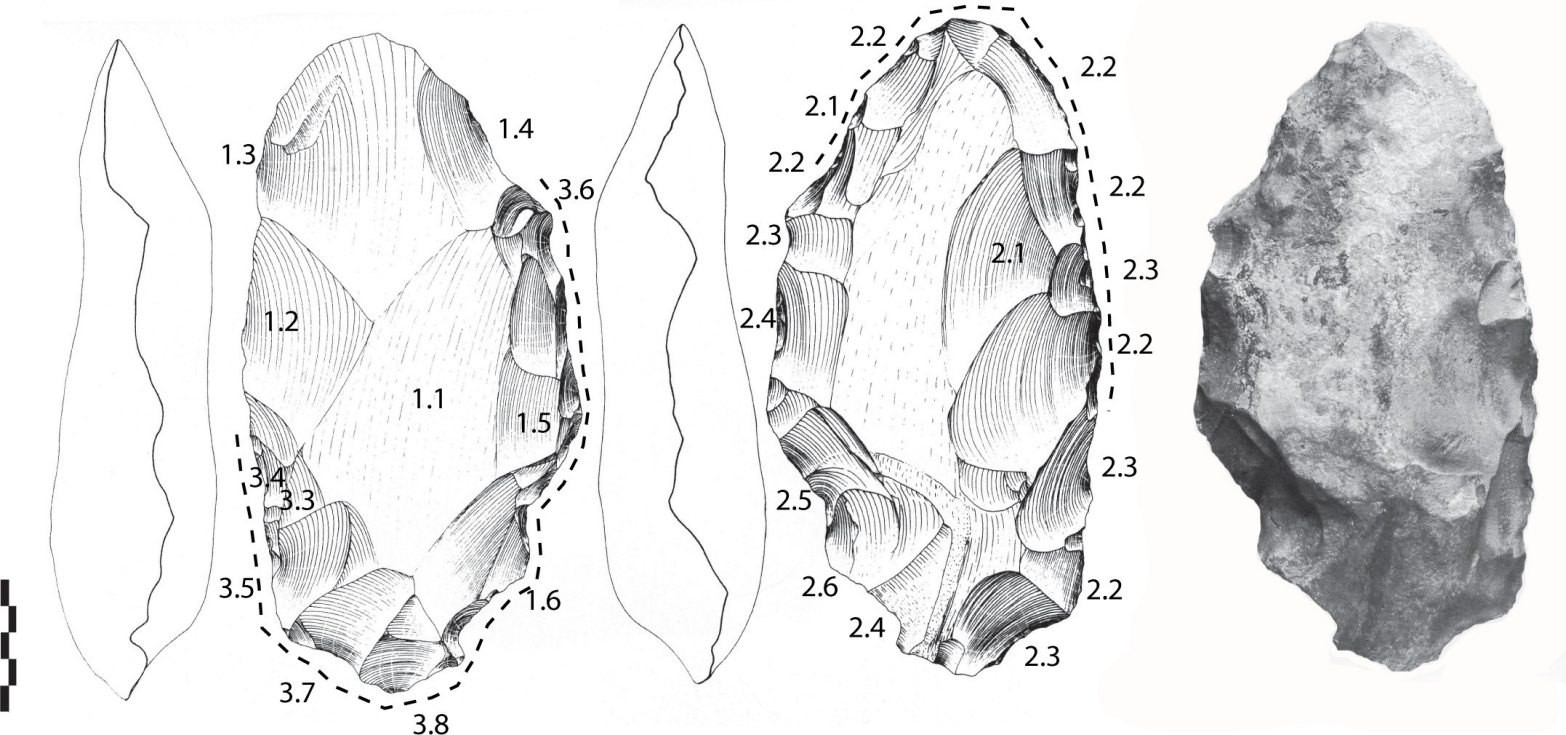
Notarchirico Archaeosurface B. Flint biface. Dotted line: final removals or retouch. The numbers indicate shaping phases and sub-phases (drawings M. Pennachioni, modified; photo L. di Masi).

The largest biface of the series and the site is on a large flint nodule/cobble with flat cortical surfaces (220-120-48 mm) (Fig 7). Shaping covers one whole surface with invasive and flat removals, then sporadic alternate small removals extend over half the face. After that, the second face was minimally worked by peripheral and shorter/deep removals. The biface is oval-shaped and the cross-section is symmetrical with sinuous edges.

The third biface in flint (149-100-45 mm) was possibly manufactured on a large flake (Kombewa or natural?) with minimal bifacial shaping on the periphery aiming to work the edges and the tip on one face, taking advantage of the natural form of the blank and the flat inferior face (Fig 6A). The tip is pointed and was finished by small and abrupt removals. This blank was selected for its general shape in order to produce a tool with two convergent edges and a pointed tip.

The angles of the cutting edges vary between 70 and 88° for the three bifaces.

Limestone LCTs (n = 2). The tools are on thin and biconvex rounded cobbles (Fig 6C and 6D), or on a large flake/broken cobble for one (Fig 6D). They are characterized by an oval shape, in contrast to the more elongated bifaces in flint (134-122-98 mm; 101-99-52 mm). Shaping covers most of the two faces with a series of large centripetal or crossed removals, then partial small removals on the edges. The shaping mode follows the geometry of the cobble. For one (Fig 6C), the small removals or retouch are unifacial on the two faces. The tip is round or transversal and not clearly retouched. The cross-section is symmetrical with cutting edge angles between 80 and 85°.

#### Archaeosurface D (n = 2)

This level only yielded two bifaces; one in flint and the other in limestone.

**Fig 8:**
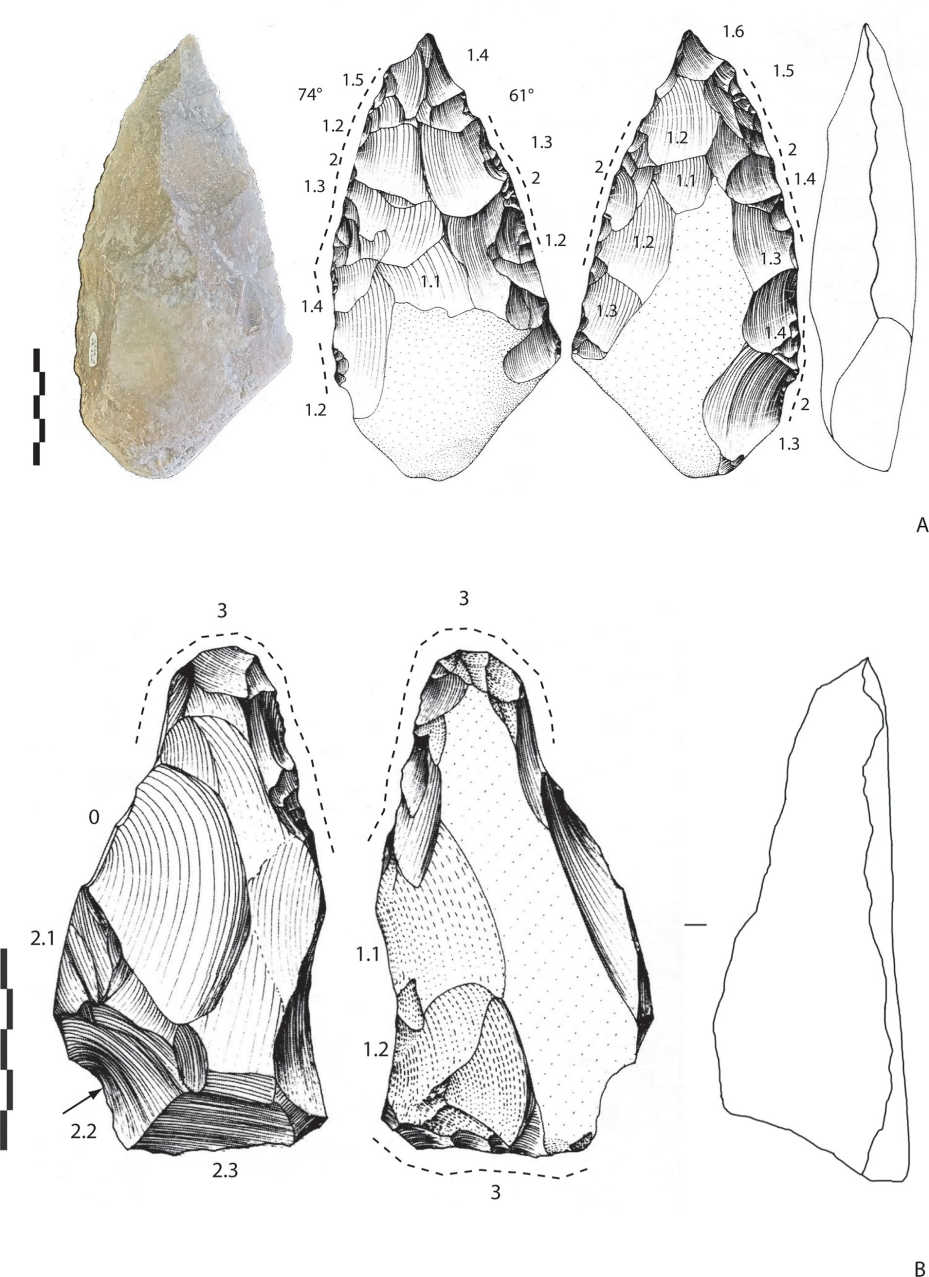
Notarchirico. Archaeosurface D. A. Biface on flint cobble. B. Biface on irregular limestone cobble. Dotted line: final removals or retouch. The numbers indicate shaping phases and sub-phases (drawings M. Pennachioni, modified; photo C. Santagata, M-H. Moncel).

The flint biface is on a flat and thin cobble (181-90-46 mm) (Fig 8A) with a preserved cortical base. Shaping is invasive on one face but is more limited to the edges and the apical part on the opposite face. The cross-section is symmetrical. Shaping is managed by large alternate removals, followed by small removals/retouch. The edge angles are 61 and 74°. The tip is pointed, ended by small removals/retouch (which look like notches).

The limestone biface is on a plano-convex and irregular cobble (138-70-49 mm) (Fig 8B). The natural shape is preserved by shaping, which takes advantage of the original form and flat surfaces. Flat, thin and invasive removals cover one face while the opposite face was shaped by abrupt removals, followed by retouch/small removals on the rounded tip. The tool base is prepared by abrupt removals. The angles of the cutting edges vary between 66 and 90°.

#### Archaeosurface F (n = 9)

**Fig 9:**
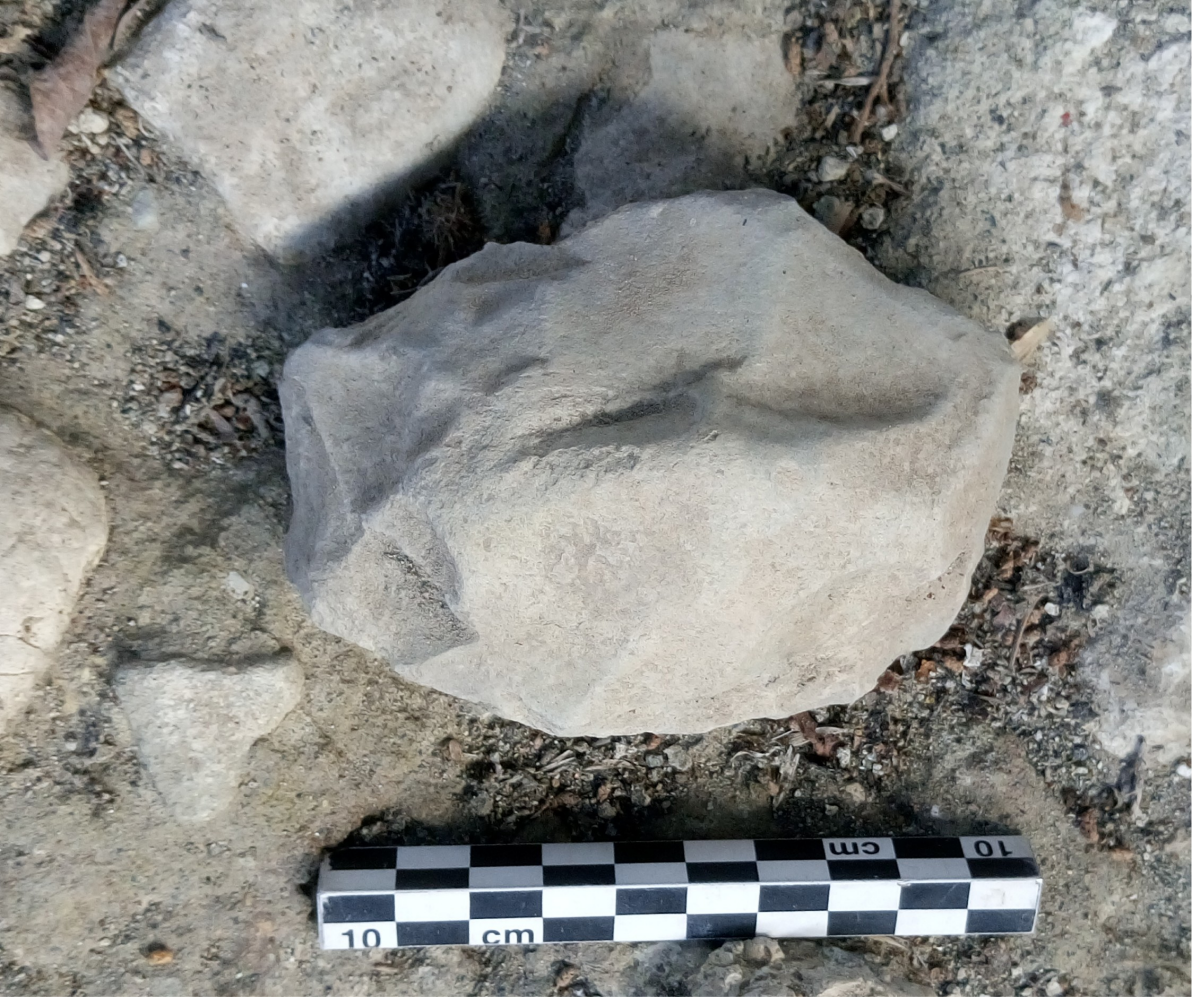
Notarchirico Archaeosurface F. Badly-preserved limestone biface, *in situ* on excavations. (photo C. Santagata, M-H. Moncel).

Limestone LCTs (n = 7). Three bifaces are badly preserved, small (71-48-36 mm) or large (124-78-44 mm), made by a series of large face-by-face removals (Fig 9). Large bifacial removals and short removals are only visible on one face. A lateral back is preserved. The tip is sometimes broken. One of the bifaces has a broken tip (111-60-51 mm). One face is shaped by large then short removals while the opposite face is shaped by abrupt and peripheral removals.

The best-preserved bifaces are described below.

**Fig 10:**
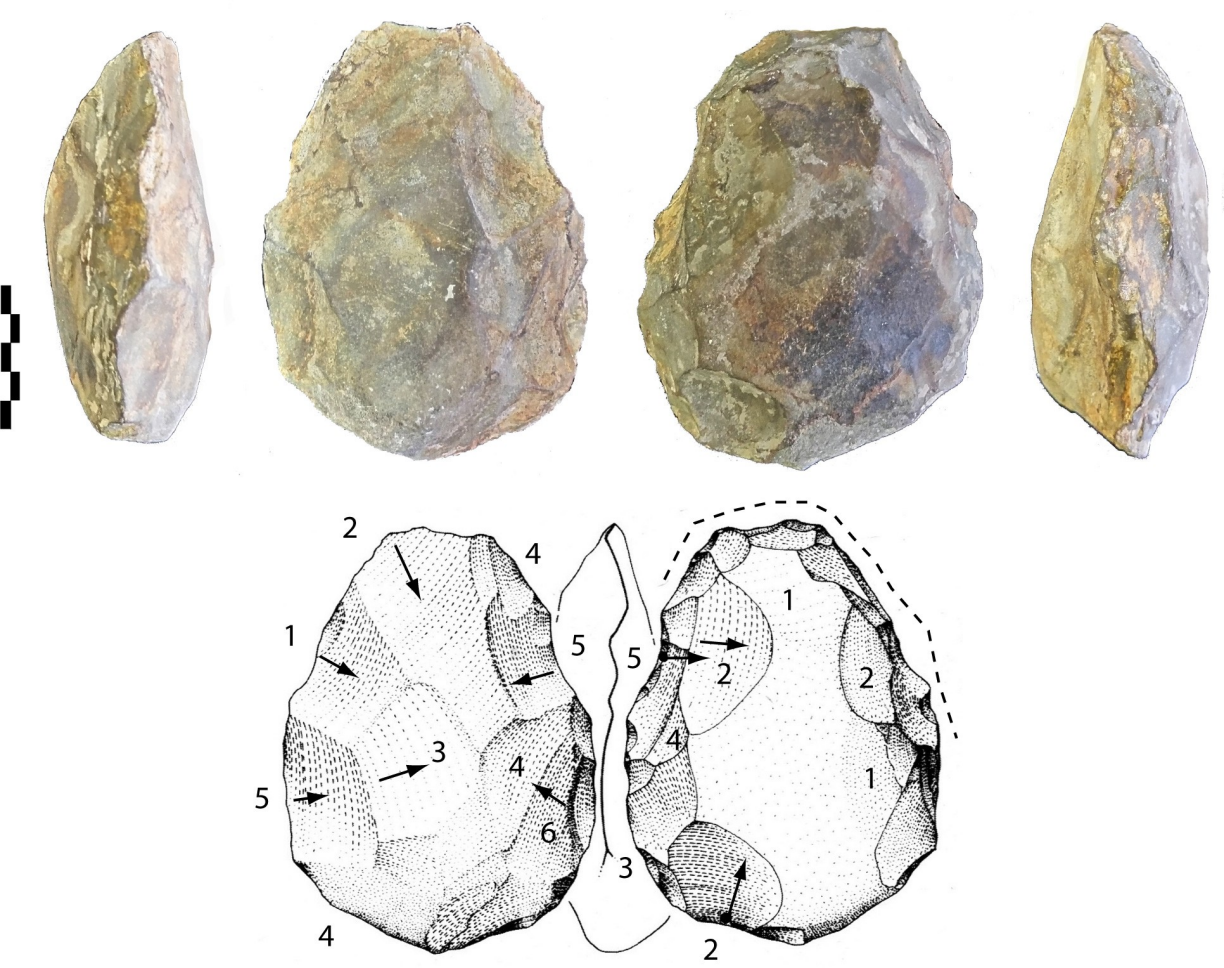
Notarchirico Archaeosurface F. Biface on biconvex limestone cobble. Dotted line: final removals or retouch. The numbers indicate shaping phases and sub-phases. Black arrows: direction of the removals (drawings M. Pennachioni, modified; photos C. Santagata, M-H. Moncel).

-The first tool is on a biconvex cobble (127-96-48 mm). Shaping covers one face by a series of large and invasive removals whereas the opposite face was shaped by smaller and more abrupt removals (less than 6) (Fig 10). The section is partially symmetrical and the edges are sinuous. The tip is round, made first by a single invasive removal and finished by short abrupt removals on one side. The overall shaping is alternate.

**Fig 11:**
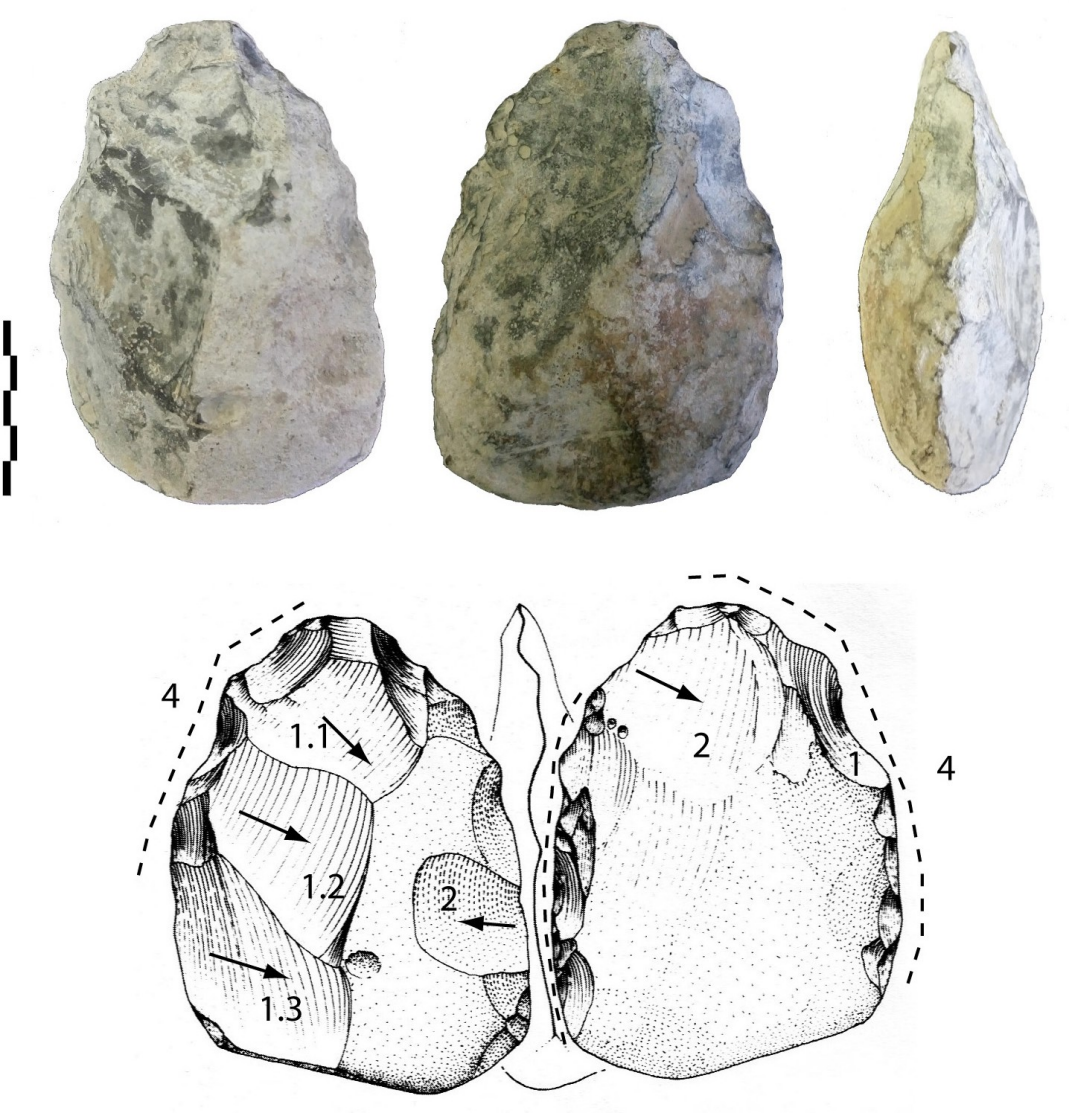
Notarchirico Archaeosurface F. Biface on biconvex limestone cobble. Dotted line: final removals or retouch. The numbers indicate shaping phases and sub-phases (drawings M. Pennachioni, modified; photos C. Santagata, M-H. Moncel,).

-Another tool is on a biconvex cobble (116-86-37 mm) (Fig 11). Shaping is limited to the upper half of the tool, the edges (85–90°) and the tip. However, a series of large removals manages the volume with some short final bifacial removals on the edges.

**Fig 12:**
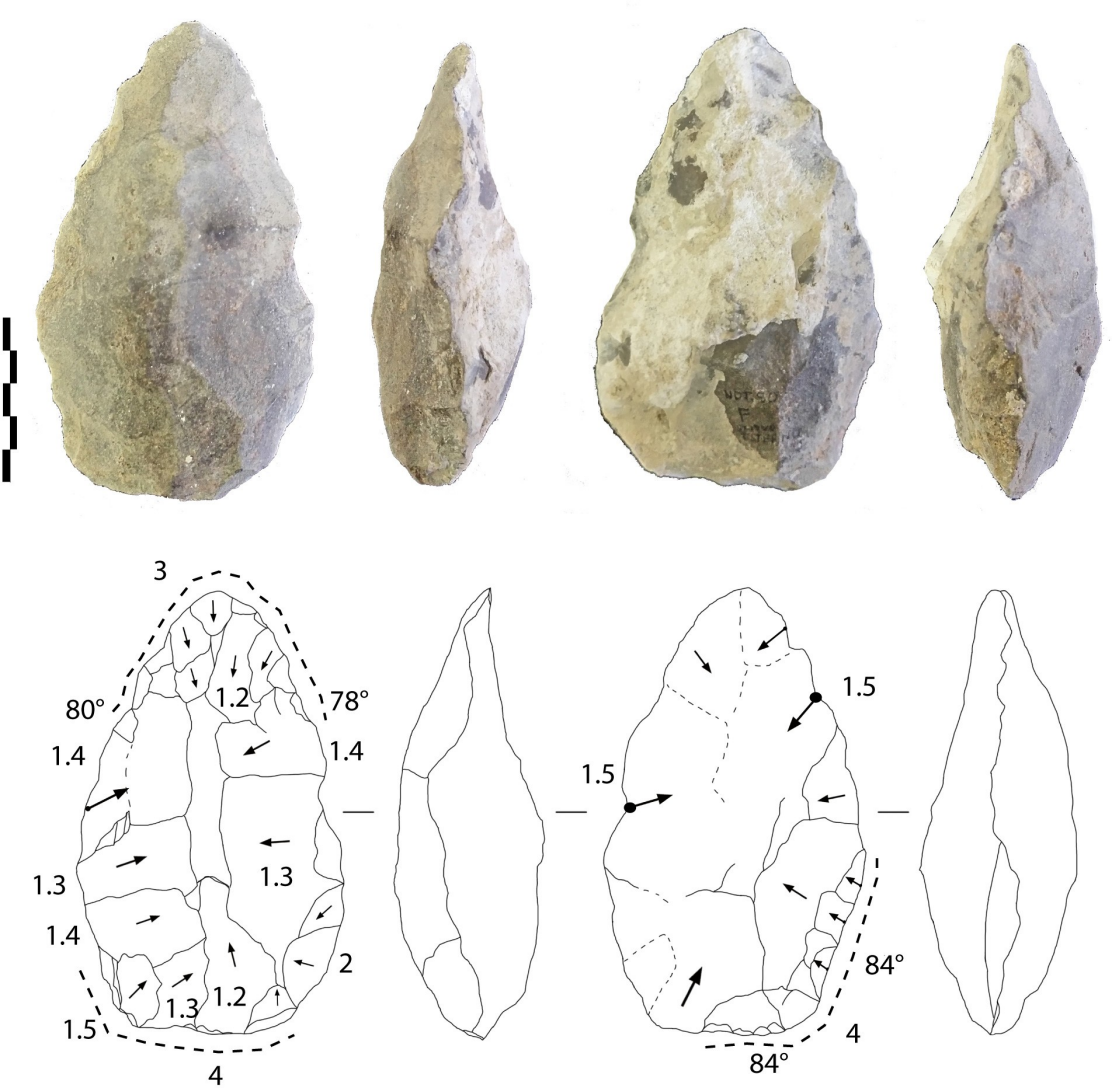
Notarchirico Archaeosurface F. Limestone biface (with breccia partly covering one face) Dotted line: final removals or retouch. The numbers indicate shaping phases and sub-phases. (drawings M-H. Moncel; photos C. Santagata, M-H. Moncel,).

-An almost entirely shaped elongated and pointed tool (142-89-58 mm) bears no cortex (Fig 12). Despite breccia on one face, large removals, some of which are hinged, cover both faces and manage the general volume and equilibrium of the tool. Short removals end the shaping of the upper part of the tool and the base. Shaping seems to be alternate, using available angles and surfaces. The edges (78–84°) are sinuous. The cross-section of the biface is symmetrical, with maximum thickness located in the middle of the piece.

**Fig 13:**
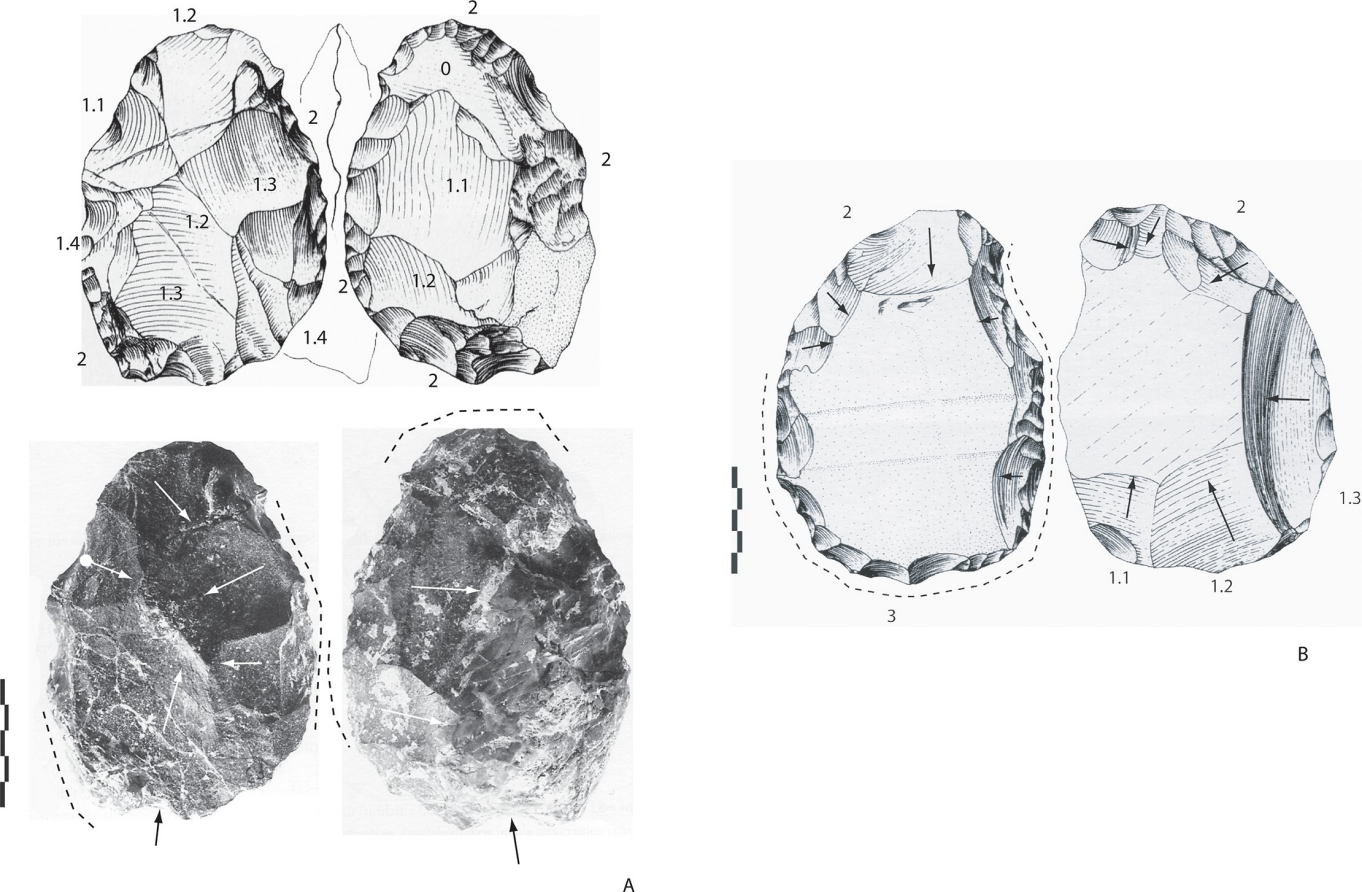
Notarchirico A. Archaeosurface F. Quartzite biface. B. Archaeosurface F. Quartzite biface. Dotted line: final removals or retouch. The numbers indicate shaping phases and sub-phases. (drawings M. Pennachioni, modified; photos L. di Masi, modified).

Quartzite LCTs (n = 2). The volume of the first tool is totally shaped by invasive removals and alternate shaping preparing the general volume (110-70-39 mm) (Fig 13A). On one face, removals are centripetal, while on the opposite face, they are crossed. Then, short peripheral removals regularize the edges and the round tip, which is the thinnest part of the tool. The base is worked by separate abrupt removals (and a notch or one abrupt removal). If we consider the short removals, three areas are visible, the apical part, one edge and a lateral side of the base, which can be explained by the final shaping or the intention to prepare specific active areas on the biface (bifacial tools by additional functional areas rather than a biface with general volume management?). The upper half of the tool is symmetrical.

The second tool is possibly a large flake (148-100-45 mm), which explains the limited but peripheral shaping of the edges (peripheral, denticulate and abrupt removals) and the tip (Fig 13B). The removals are flat, more invasive and transversal on the round tip (resharpening of the broken apical part?). The inferior face is thinned by flat and invasive removals located at the base of the tool. Despite limited shaping, this tool was included in our corpus on account of the general design, aiming to manage the whole periphery of the blank. However, this piece could also be considered as a large bifacial peripheral scraper.

### Bifacial strategies at Notarchirico

The new technological analysis of the bifaces recovered from the sequence at Notarchirico can be summarized as follows:

- The number of LCTs (bifaces and bifacial tools for some) is always low (see Table 2). They are associated with a large quantity of pebble tools for levels A1, B and D, but not for level F (mainly using the most widely available raw material, limestone). Except for level F, local flint was also used when the nodules were large enough.

- Shaping left residual cortical patches, often on the base, except for four pieces, facilitating the identification of the type of blank. When identifiable, blanks are mainly thin, flat or biconvex whole or broken cobbles, with some rare large flakes (≥ 10 cm) (n = 2 or 3 on archaeosurfaces B and F). The geometry of the blanks suggests that they were carefully selected, and circumvents the primary shaping phases of the tool in some cases. Due to the absence of giant cores and the paucity of unmodified large flakes on the site, we cannot determine the core technology used for producing the rare large flakes. The reduction sequence may have been fragmented (off-site production of large flakes), and the large flakes or already worked tools may have been brought from outside or moved from other parts of the site. The biface shaping process is thus independent of the core technology process at the site. This observation has important implications for understanding hominin mobility, site function and occupation intensity. If large flakes were already knapped before being brought to the site, we can assume that hominins planned their occupations before arriving on the lakeshores/river shores and discarded their large and small tool kits when they left. We should also keep in mind that the occupation area may have been much larger in size, and flakes and tools may have been moved around depending on activities. Large flakes could have been produced elsewhere on the site. Each archaeological record is only a narrow window of the occupations. Raw material sources were local, selected from the beds of cobbles and pebbles [30].

- For bifacial shaping, large removals form the first full bifacial volume for flint and cover the surfaces of the tool, except for four pieces with limited shaping extension (bifacial tools v. bifaces?). Removals are shorter in some cases for limestone and quartzite cobbles, and often confined to a single series. Then shorter removals/retouch shape the edges and/or the upper half of the flint and some limestone and siliceous limestone tools. The structure of shaping does not distinguish techno-functional units but indicates general volume management for optimal bifacial and bilateral symmetry.

-The shaping mode is alternate, except for five pieces (face-by-face) and takes advantage of the available angles and surfaces of previous removals, indicating the management of the whole blank volume and bifacial equilibrium, regardless of the type of stone and blank geometry. Shaping is not restricted to the edges, and when the extension of removals is limited (n = 5), this is generally due to the geometry of the blank (large flake or thin and biconvex cobbles, for instance).

**Fig 14:**
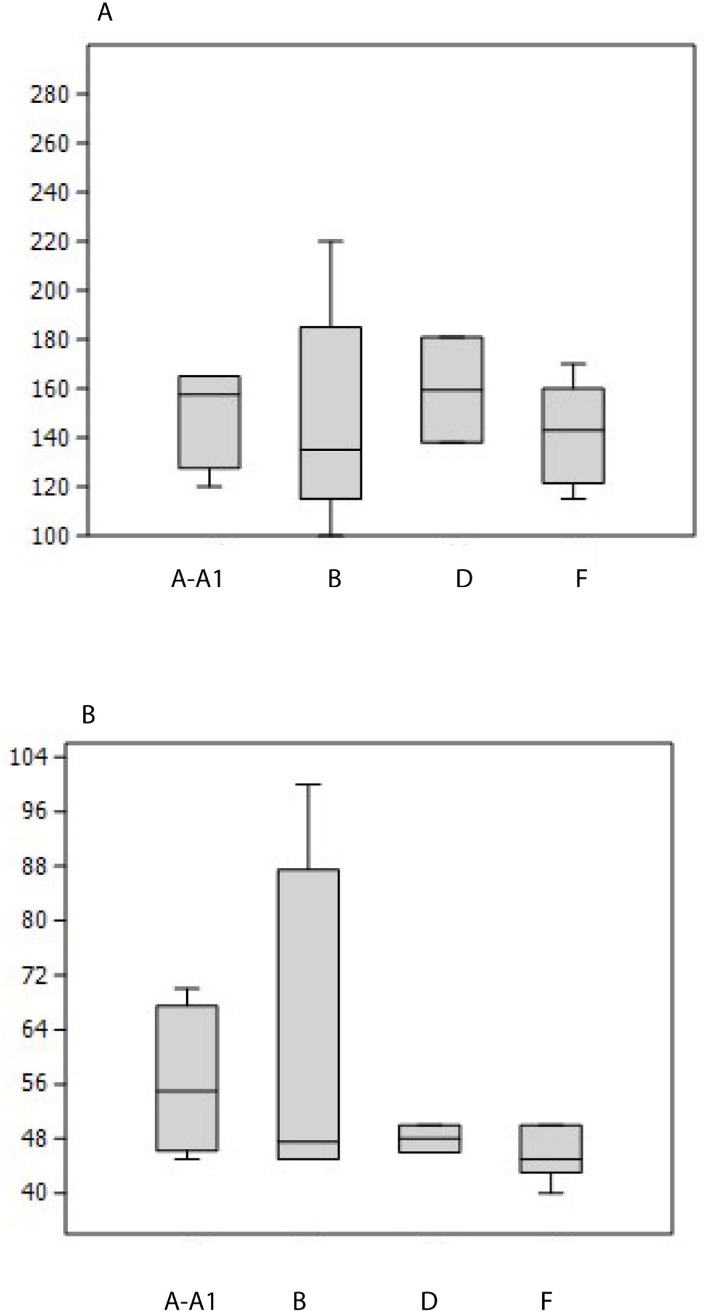
Notarchirico. A. Maximum length in mm of bifaces by archaeosurface. B. Maximum thickness in mm of bifaces by archaeosurface.

**Fig 15:**
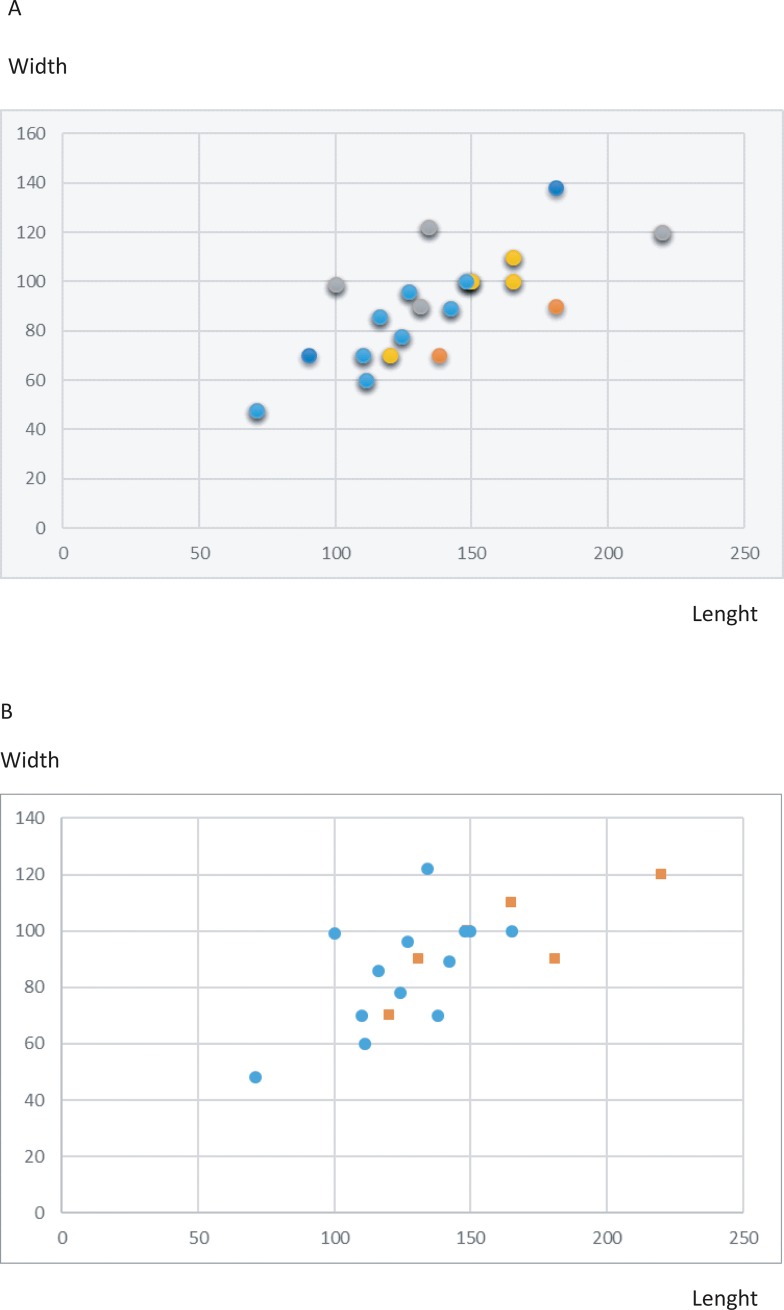
Notarchirico A. Graph Length/width (mm) of bifaces by archaeosurface. Yellow: A-A1: Grey: B, Orange: D, Blue: F. B. Graph Length/width (mm) of bifaces by raw materials. Orange squares: flintBlue circles: limestone and quartzite.

- Most of the tools are bilaterally and bifacially symmetrical forms (70 and 85% respectively), irrespective of the intensity of shaping, and the type of blank and stone.

- Tool maintenance (resharpening phases) is not clearly visible on bifaces with intensive shaping. A few tools from levels B and F (n = 4) with a deep removal followed by some retouch or transversal removals do not constitute clear evidence of resharpening, as this may be part of initial tool shaping. For tools with one series of removals (irrespective of the number of removals), we can assume that shaping corresponds to the initial reduction phase (see Figs 8, 10 and 11). Shaping intensity is not related to the inferred distance from raw material sources (for the flakes). The largest shaped tools are on limestone and flint cobbles available on the lakeshore.

- The strategies used indicate that the impact of the type of stone was limited but nonetheless present. We observe more intense shaping and more removals on flint or siliceous stones, due perhaps to the quality of the stone. However, some limestone bifaces are also intensively shaped. The original geometry of the blank cannot always account for the intensity and mode of shaping.

- The presence of hinged removals on eight tools suggests poor management of the angles during the final shaping phase. The angle values of the large and short removals and the depth of the scars on both faces suggest that only hard hammers were used for shaping, which could explain the hinged removals at the end. This could also account for the difficulties in identifying flakes issued from shaping. The absence of thin and curved flakes with a lip in the assemblages also tends to rule out the use of a soft hammer [9]. Except for small flint flakes produced by cores on small nodules, the rest of the assemblages is composed of medium-sized thick flakes related both to knapping and shaping processes.

- Cross-sections and forms are symmetrical or asymmetrical, either as a result of the natural shape of the blank or the mode and intensity of shaping. Final retouch does not accentuate tool symmetry or asymmetry but helps to improve the regularity of the cutting edges, especially on flint.

- Cutting edges are often sinuous but are more regular for flint, and for several bifaces they extend around the whole periphery of the tool. Cutting edge angles vary along the periphery (generally 75–85°, with some lower angles for flint) but the angle of the tip is always the lowest.

- The tip is pointed or round, with final retouch/small removals for ten tools, whereas the base of the tool is frequently worked by additional (and abrupt) removals in some cases, some of which are retouched. These additional removals can be considered as evidence of further functional tool parts *i*.*e*. [43], or as part of general bifacial management. Primary shaping of the tip is generally part of overall tool shaping. Some tools show one or several transversal removals aiming to create a rounded or transversal extremity.

- The comparison of the maximum length and thickness of the bifaces by level does not indicate a clear difference between the series, but the number of pieces is limited, especially for level D. Tool size is more homogeneous in level F, the largest series (Figs 14 and 15). Tools are generally rather short and thick (> 50–60 mm) (Fig 15). However, some tools are large and elongated (> 150–165 mm) and only one biface is backed. The diversity of biface size cannot be explained by shaping mode and intensity. The smallest pieces do not correspond to tools reduced in size by shaping and there is no size continuum in relation to the type of biface production.

## Discussion: What about biface shaping in Western Europe between 700 and 600 ka?

### Cognition and skills of Middle Pleistocene hominins between 700 and 600 ka in Western Europe

The technological analysis of the bifaces from Notarchirico clearly shows that hominins had the capacity to manage bifacial volumes by alternate or face-by-face shaping during glacial stage MIS 16 (676–621 ka), when raw materials were of sufficient quality. Several series of removals contributed to rectifying the edges and the tip. The tools present sinuous edges despite the sporadic use of final retouch, on account of the use of limestone pebbles instead of flint nodules. We observed a mixture of bifaces *sensu stricto* and cruder bifacial tools depending on the extension of shaping. Clear differences do not emerge between the different levels in terms of shaping modes or final shapes and cutting edge angles. However, we noted that the oldest level (level F, < 670 ka), with the richest corpus, contains few flint artefacts and a higher diversity of biface shaping modes and morphological results, but with limited variations in length and thickness. Conversely, level B at the top of the sequence yields the highest quantity of crude bifacial tools in flint and limestone and the highest diversity of sizes (length and thickness) for the series. In levels A, B and D, bifaces and bifacial tools are associated with pointed unifacial and bifacial pebble tools. The shaping of these pebble tools is limited to one or two edges and the tip on thick pebbles, and the tip is not rectified by additional removals. The minimal shaping mode is completely different to that of bifaces/ bifacial tools and crude bifacial tools. The pointed pebble tools are not stages in a single shaping reduction process (see Fig 2). Should these pebble tools be considered as the result of shaping adaptation to diversified pebble shape or merely as the basic strategy required to produce pointed tools in different ways (for diverse activities or in keeping with traditions)? In both cases, they show that the shaping processes and technological strategies of Middle Pleistocene populations were flexible, *e*.*g*. [44].

The high quantity of bifaces for the oldest level F, with a limited excavated area and low number of pebble tools, raises questions regarding the nature of occupations. The association between artefacts and bones is not clearly demonstrated in the detailed analysis of the “Elephant Butchery area” at the top of the sequence (archaeosurfaces A-A1-B) [19, 45]. The reason underlying the presence of six crude bifacial tools in level B is also difficult to explain, as is the lack of bifaces in levels C and E. No clear difference in technological strategies can be retained to explain differences in bifacial shaping in the occupation layers. However, the disparate surfaces of the excavated areas in different levels could account for some particularities and divergent counts. Core technology is similar to core-and-flake assemblages such as Gran Dolina, TD6 level (Atapuerca) or Vallparadís in Spain, prior to 780 ka [18, 46], unlike penecontemporaneous sites, which record some knapping innovations. The site of Isernia la Pineta (Italy, Late MIS16, ≈ 580 ka) [47] did not yield LCTs but contained a large quantity of pebble tools with a huge quantity of herbivore carcasses [10, 48]. The lithic assemblage from level t.3c indicates that the small debitage was opportunistic. It is similar to debitage at la Noira site with some structured flaking modes regardless of blank geometry [9].

This ability to manage bifacial and bilateral equilibrium, as well as the diversity of the morphological results (shapes, cutting edge angles, length of available edges, tip shapes, management of the base), are also observed in the few known penecontemporaneous sites in the north-western regions of Western Europe, between 700 and 600 ka: at la Noira (Early MIS16, 650–700 ka), located in the Centre Region of France, as well as Moulin Quignon (Early MIS16, ≈ 650 ka) in north-western France and at the slightly younger Brandon Fields site (MIS 15/14, ≈ 560 ka) in the U.K. [4, 5, 6, 9, 25, 49–56]. The technological study of the bifaces of Notarchirico shows that this site is not an isolated case and it signifies that it is not a persistent Mode 1 type site or evidence of local evolution. The features of biface production (shaping mode, number, size and organization of the removals, tool equilibrium by volume management) indicate the control of this technology and suggest a technological shift in comparision to the Mode 1 type sites. The ability to manage bifacial volume is therefore observed in populations occupying Southern Europe from 675 ka onwards, suggesting that common technological behaviour extended over the whole of Western Europe at the same period.

For sites with LCTs, diverse tool blanks were selected (slabs, cobbles, nodules or fragments), irrespective of raw materials, along with a few large flakes. This does not mean that European hominins were not capable of producing this kind of blank at 700–600 ka, or of managing stone procurement and territories (for instance, large flakes produced at outcrops). It simply indicates selection and adaptation to local stones and geometries directly available in situ.

At la Noira, we observe large flake production with some giant cores on slabs [25, 57], while at Notarchirico, giant cores are absent and only a few limestone chopper-cores were intended to produce larger flakes. No comparable artefacts were observed in quartzite and flint. Large flake production may have been fragmented. The few large flakes (50–100 mm long) at Notarchirico are unretouched or retouched (denticulate or scraper-type) and the rare bifaces on flakes are on the largest flakes. At la Noira, large flakes were produced *in situ*, but this type of blank was rarely selected for making a biface or a LCT in general. For the British sites, local flint nodules of various sizes were available in abundance, possibly explaining the predominant use of whole nodules rather than large flakes.

Intra-site variability is also a marked feature of these series. At la Noira (France), the 61 heavy-duty tools found among the artefacts (total = 915) include bifaces (n = 8; 97–228 mm long) [25]. Besides bifacial tools, which are characterized by edge modifications only and look like large bifacial convergent scrapers, some bifaces show full volume management. They are made on slabs and fragments of limestone slabs, rarely on large flakes. The cross-section is often asymmetrical (plano-convex), regardless of the shaping mode [54]. They are made by series of multiple invasive and short removals, and final retouch prepares the cutting edges and the pointed tip. They all bear traces of hard hammer percussion, although a soft hammer could have been used for some final phases (thin and invasive curved removals at la Noira). At Brandon Fields (UK), bifaces are generally bifacially made with a hard hammer on flint nodules/pebbles, half-pebbles, and again, occasionally on flakes, and are of variable size [7, 12]. They are elongated or short (58 to 167 mm long), thin or thick and crudely shaped, often resulting in sinuous cutting edges, with pointed tips and cortical butts. Shaping methods are alternate or face-by-face, with little final retouch or evidence of resharpening. Some have plano-convex cross-sections due to the use of split pebble blanks or to shaping with a limited number of removals. The result is a mix of morphological and technological types with bifaces *sensu stricto* and cruder bifacial tools. There is little evidence of soft hammer working. The assemblages appear to reflect a more basic knowledge of biface technology, in marked contrast to the more elaborate systems used on some tools at la Noira or Notarchirico.

These common patterns suggest that hominins mastered well-controlled and diversified biface production as early as 700 ka, with intense shaping and minimal shaping side by side in the assemblages. They appear to have shared a common technological approach regardless of the geographical area and applied this technology irrespectively of the available raw materials. The function of bifaces does not explain this intra-site diversity, as demonstrated by [58] for la Noira, where various types of residues on LCTs suggest that they were multi-functional tools. On the other hand, inter-site diversity may be related to site function, varied traditions, cognition or different hand gripping abilities, as suggested by [59]. Only Notarchirico yielded a hominin femur fragment and so far, we have no idea of the hand morphology of hominins occupying Western-Europe between 700 and 600 ka. This biface-making ability persists during MIS 14–13, although some tools became more standardized, for instance at la Caune de l’Arago (levels P-Q) [60, 61], and particularly at Boxgrove [62, 63].

### A cultural shift in Western Europe at 700–600 ka?

This technological revision of the Notarchirico bifaces shows that the 700–600 thousand-year-old series of European bifaces share common technological approaches, in all geographical areas. Hominids already mastered bifacial tool volume, and biface production is documented in different areas of Western Europe. These tools display varying degrees of shaping intensity and diverse morphological results, which cannot be explained solely in terms of raw material quality. This ability is either associated with innovations in core technologies (independently of stone geometry) and/or land use (fragmentation of large flake production).

It is important to note that the bifacial tool kit in Europe was not standardized at that time, while contemporaneous assemblages in East Africa at Gombore II (Ethiopia) [64, 65] or Isenya (Kenya) [37] and in the Levant at Gesher Benot Yakov (GBY) (Israël) [39, 66, 67], indicate higher standardization. This may be due to the small number of bifaces for each site in Europe, while in East Africa and the Levant, the series often contain higher numbers of bifaces. In East Africa, the archaeological record differs. An Early Acheulean from 1.75 Ma is followed by a classical/Middle Acheulean from 1 Ma/800 ka to 600 ka [37, 68]. We observe marked technological and economical discontinuity with more intense shaping of standardized LCTs and the widespread use of large flakes as blanks from 1 Ma to 800 ka. We can also mention inter- and intra-site variability, the use of soft hammers and the fragmentation of reduction processes.

This dichotomy between lithic records in East Africa, the Levant and Western Europe between 1 Ma and 500 ka raises questions concerning the diffusion or the local origin of biface production in Eurasia [4, 5, 6, 14, 65]. Archaeological data suggest a technological shift at 700 ka with the introduction of new technologies, including bifacial technology, episodically associated with innovations in core technologies and land use. Bifacial shaping extended over the whole of Western Europe during the same narrow chronological window. We do not see any evidence of a south to north-west expansion, except in the UK, in younger lithic series with bifaces. Clear evidence of Acheulean diffusion in the Levant from Africa is recorded at Ubeidiya, then at GBY (from 1.4–1.2 Ma to 900 ka), and also in India and Southeast Asia, where Acheulean bifaces are documented between 1.5 Ma and 800 ka [4–6, 39, 69, 70]. The inter- and intra-site diversity of LCTs and the infrequent use of large flakes in Western Europe at 700–600 ka could be due to the type of management of available raw materials and/or occupation types with limited hominin mobility [71]. Cut marks and fragmented bones demonstrate meat processing or butchery on carcasses of herbivores of different sizes, including elephants and other large herbivores, at penecontemporaneous or younger sites [72–74]. Sites seem to be either multi-activity short or long-term occupations or specialized sites (possibly butchery at Isernia-La-Pineta, Ficoncella, Cimitero di Atella in Italy; Boxgrove in UK) [72–78]. They suggest mobile groups in a territory composed of well-known places, using local raw materials with little evidence of semi-local procurement of stones [10, 77, 79, 80]. The more recent site of Caune de l’Arago is an exception with the use of some long-distance stones from a territory of 40 km [60].

The diversity in shape and shaping modes of bifaces in Western Europe at 700–600 ka also raises questions regarding group size and the number of knappers capable of making these tools (see the hypothesis of experts at GBY in the Levant from 900 ka [66, 81]). Moreover, the lack of cleavers on flakes and bifacial cleavers at Notarchirico, which are present in small quantities at la Noira, suggests the possible existence of multiple traditions in Europe due to raw material constraints, sparse non-connected groups and/or phases of colonization and depopulation between MIS 16 and 12.

Sharon [82, 83] describes the expansion of Large Flake Assemblages (LFA) from East Africa to the Levant from c. 900 ka (as attested at GBY), and to Asia. These LFA assemblages are characterized by the predominant use of large flakes for LCT production. The LFA tradition did not seem to have reached Western Europe at 700–600 ka, unless the primary adaptation of populations to new environments led to the limited use of large flakes for LCT production [84].

## Conclusion

The technological analysis of 32 tools from the A-A1, B, D and F archaeosurfaces indicates that hominins at Notarchirico had the capacity to manage bifacial volumes, when raw material quality was adequate. Clear differences do not emerge between the different levels in terms of shaping modes or final forms. The oldest level (level F), with the richest corpus, displays a higher diversity of bifaces. The new excavations started in 2016 on the bottom of the sequence below level F (four new archaeological levels G, H, I and J) will make it possible to determine whether a clear technological shift exists at level F (670 ka) with the biface production ability.

This ability to manage bifacial and bilateral equilibrium, as well as the diversity of the morphological results, is observed in a few penecontemporaneous sites (700–600 ka), both in the northwestern and southern parts of Western Europe. These patterns suggest that hominins mastered well-controlled and diversified biface production, combining intense shaping and minimal shaping, and shared a common technological background regardless of the geographical area and the available raw materials.

The degree of complexity and the skills of hominins in Western Europe between 700 and 600 ka, the apparent lack of “gradual industries” between core-and-flake series (Mode 1) and Acheulean (Mode 2) techno-complexes, such as in East Africa (Early Acheulean) [14, 85, 86], as well as the presence of already elaborate Acheulean techno-complexes as early as 700 ka, raise numerous questions on the origin of new behaviours in Western Europe, their mode of diffusion, and their association with *Homo heidelbergensis* or Middle Pleistocene populations [8, 87–89].

The specific period of interest here is the end of the ‘Middle Pleistocene Transition’ (MPT), and especially from 700 to 450 ka, before the MIS 12 glacial event. Cyclical climate change could have led to the successive depopulation or extinction of small groups of hominins, and subsequent recolonization through opening or closing coastal corridors, conducive to expansion from the Levant and Africa [90–94]. Dennell et al. [24] suggest successive phases of recolonization from Asia (“ebb and flow processes” or a “sink-source model”), with evolutionary processes in refugia. Recent discoveries of early sites in Greece indicate possible routes along the Mediterranean coast [95, 96] and crossing the Strait of Gibraltar cannot be totally ruled out [83], especially as it has been demonstrated that early hominids populated the island of Luzon more than 700 ka ago [97]. The most propitious occupation periods seem to be transitional phases between interglacial and glacial episodes, with the opening of landscapes before the arrival of the cold/drier period in north-western Europe and warmer and more humid conditions in Central Europe [98]. These environmental changes could have promoted episodic hominin expansion across Western Europe, from the northwest and the south, aided by new techniques, new social organizations, with better access to prey (often megaherbivores) in mid-low latitudes and adaptation to European ecosystems (more energy, richer food sources), *e*.*g*. [99–104]. This hominin expansion would correspond to the colonization of Europe either by *Homo heidelbergensis*, or by a mixture of new Middle Pleistocene hominins whose origin remains enigmatic, as suggested by [8, 87–89, 105].
