# Correction: A biface production older than 600 ka ago at Notarchirico (Southern Italy) contribution to understanding early Acheulean cognition and skills in Europe

**DOI:** 10.1371/journal.pone.0224603

**Published:** 2019-10-24

**Authors:** Marie-Hélène Moncel, Carmen Santagata, Alison Pereira, Sébastien Nomade, Jean-Jacques Bahain, Pierre Voinchet, Marcello Piperno

There are errors in the Author Contributions. The correct contributions are:

**Conceptualization:** Marie-Hélène Moncel

**Data curation:** Alison Pereira, Sébastien Nomade, Jean-Jacques Bahain, Pierre Voinchet, Marcello Piperno

**Formal analysis:** Marie-Hélène Moncel, Carmen Santagata, Alison Pereira, Sébastien Nomade, Jean-Jacques Bahain, Pierre Voinchet

**Funding acquisition:** Marie-Hélène Moncel

**Investigation:** Marie-Hélène Moncel, Carmen Santagata, Alison Pereira, Sébastien Nomade, Jean-Jacques Bahain, Pierre Voinchet

**Methodology:** Marie-Hélène Moncel, Sébastien Nomade, Jean-Jacques Bahain, Pierre Voinchet

**Supervision:** Marie-Hélène Moncel

**Validation:** Marie-Hélène Moncel

**Writing–original draft:** Marie-Hélène Moncel, Alison Pereira, Sébastien Nomade, Jean-Jacques Bahain, Pierre Voinchet

**Writing–review & editing:** Marie-Hélène Moncel
